# Carbonic Anhydrase Inhibitor Acetazolamide Enhances CHOP Treatment Response and Stimulates Effector T-Cell Infiltration in A20/BalbC Murine B-Cell Lymphoma

**DOI:** 10.3390/ijms21145001

**Published:** 2020-07-15

**Authors:** Gábor Méhes, Orsolya Matolay, Lívia Beke, Marianna Czenke, Róbert Pórszász, Edit Mikó, Péter Bai, Ervin Berényi, György Trencsényi

**Affiliations:** 1 Department of Pathology, Faculty of Medicine, University of Debrecen, 4032 Debrecen, Hungary; orsolya.matolay@med.unideb.hu (O.M.); beke.livia@med.unideb.hu (L.B.); marianna.czenke@med.unideb.hu (M.C.); 2 Department of Pharmacology and Pharmacotherapy, University of Debrecen, 4032 Debrecen, Hungary; robert.porszasz@gmail.com; 3 Department of Medical Chemistry, University of Debrecen, 4032 Debrecen, Hungary; miko.edit@med.unideb.hu (E.M.); baip@med.unideb.hu (P.B.); 4 MTA-DE Lendület Laboratory of Cellular Metabolism, University of Debrecen, 4032 Debrecen, Hungary; 5 Research Center for Molecular Medicine, University of Debrecen, 4032 Debrecen, Hungary; 6 Department of Medical Imaging, University of Debrecen, 4032 Debrecen, Hungary; ervin.berenyi@gmail.com (E.B.); trencsenyi.gyorgy@med.unideb.hu (G.T.)

**Keywords:** lymphoma, chemotherapy, pH-regulation, adaptation, anti-tumor immunity, effector T-cells, carbonic anhydrase

## Abstract

The inhibition of cancer-related carbonic anhydrase (CA) activity is a promising way to intensify anti-tumor responses. In vitro data suggest improved efficacy of cytotoxic drugs in combination with CA-inhibitors in several cancer types. Despite accumulating data on CA-expression, experimental or clinical studies towards B-cell lymphoma therapy are missing. We therefore decided to test the effect of the CA-inhibitor acetazolamide (AA) on the conventional CHOP treatment regimen using the A20/BalbC in vivo syngeneic mouse lymphoma model. Tumor growth characteristics, 18F-MISO-PET activity, histomorphology, cell proliferation, and T-cell immune infiltrate were determined following single or multiple dose combinations. All results point to a significant increase in the anti-tumor effect of CHOP+AA combinations compared with the untreated controls or with the single CHOP or AA treatments. CD3+ and CD8+ T-cell immune infiltrate increased 3–4 times following CHOP+AA combination compared with the classical CHOP protocol. In conclusion, CA-inhibitor AA seems to act synergistically with the anti-tumor treatment CHOP in aggressive lymphoma. Further to a cytotoxic effect, AA and other more selective blockers potentially support tumor-associated immune responses through the modification of the microenvironment. Therefore, CA-inhibitors are promising candidates as adjuvants in support of specific immunotherapies in lymphoma and other malignancies.

## 1. Introduction

Hypoperfusion, tissue hypoxia, and consequential intracellular acidosis may occur as a result of massive neoplastic growth. However, adaptive changes are also induced in line with tumor hypoxia. Cellular response, generally mediated by hypoxia-inducible factor 1 (HIF1) results in energy-saving reprogramming, repression of anabolic activities, and of the cell cycle (cellular revertance) as well as in upregulation of protective pH neutralizing mechanisms [1,2,3]. These features give rise to biologically heterogenous intratumor compartments and improve resistance against environmental stress factors significantly interfering with anti-cancer treatments [4,5,6].

The expression of cell membrane-bound carbonic anhydrases (especially isotypes CA IX and CA XII) is induced in a significant portion of malignancies due to hypoxic stress [7]. CAs are transmembrane enzymes catalyzing the reversible hydration of carbon dioxide. Reaction product bicarbonate (HCO_3_^−^) passes the cell membrane and restores intracytoplasmic pH resulted by lactate and other metabolites [8]. By-product H^+^ consequently accumulates in the extracellular space and contributes to the acidosis of the tumor microenvironment with specific but still underestimated effect [9,10]. Dynamic upregulation of CAIX and CAXII was reported to be driven by HIF-1a in neoplastic cells suffering under hypoxia [11,12,13]. The expression of CAIX, and to a lesser degree CAXII, was associated with aggressive phenotype and unfavorable prognosis in diverse tumor types (including colon- and breast carcinoma, glioblastoma, etc.) [14].

A wide range of small molecule carbonic anhydrase inhibitors have been in clinical use for selected disorders outside the field of oncology, e.g., pulmonary hypertension, shock, hydrocephalus, glaucoma, etc. [15,16]. For this reason, there is profound knowledge regarding the dosage, pharmacokinetics, and toxicity for the prominent groups of inhibitory compounds. Currently used CA inhibitors are of non-selective effect with a considerable affinity to block tumor related isoenzyme CAIX and CAXII activity which could be specifically demonstrated in hypoxic cancer cells in vitro [17,18,19]. Moreover, the development of isoenzyme specific inhibitors for cancer therapy resulted in promising data. A selective effect on CAIX/XII was observed with the sulfonamide derivate compound SLC-0111 which was found clinically effective and progressed to clinical Phase II investigation [20]. Further to sulfonamides, coumarine-derivatives, and sulfamates, acetazolamide (AA) has been used as a prototype of CA-inhibitors and was the subject of experimental and clinical investigations [21]. A set of combination treatment options have already been suggested, applying CA inhibitors adjuvant to chemo- or radiotherapy [22,23,24].

Aggressive lymphomas, such as diffuse large B-cell lymphoma, Burkitt’s lymphoma and anaplasic large cell lymphoma, etc., belong to the malignancies with the highest cell proliferation also presenting with extensive necrotic foci [25]. The role of O_2_ gradient and relative tissue hypoxia in the maturation of lymphocytes and in the process of lymphomagenesis was established and recently discussed [26]. The role of CAIX upregulation in the pH homeostasis of experimental lymphoma models were demonstrated [27]. According to our observations, hypoxia related adaptation and CAIX (but not CAXII) overexpression is obvious in a subset of Hodgkin’s lymphomas with an adverse clinical effect [28,29]. While the metabolic heterogeneity within the lymphoma tissue is well known and can also be routinely demonstrated by in-vivo PET-imaging tools, therapeutic considerations focusing on an adaptive response are to date missing. To study the in vivo effect of pH modulating agents in lymphoma, we established a murine aggressive lymphoma model applying cultivated A20 B-cell lymphoma cells in BalbC mice. This experimental system was reported to be extremely helpful to evaluate the cytotoxic activity of specific drug combinations earlier [30]. As a major advantage, this model allows the simultaneous study of tumor progression together with tumor–host immune mechanisms as the immune system of the matched mouse strain remains virtually intact. We aimed to demonstrate the potential benefits of CA-inhibitor AA as an adjuvant agent in combination with the clinically applied standard CHOP (cyclophosphamide, hydroxydaunorubicin, oncovin/vincristine, prednisolone) lymphoma treatment regimen. In-vivo growth and metabolic activity, cell kinetic studies, and the measure of immune infiltrates were defined, and all indicated an intensified anti-tumor response of the adjuvant AA on the combined treatment.

## 2. Results

### 2.1. Effect of CHOP-AA Combination on In Vivo Tumor Metabolic Activity

**Figure 1:**
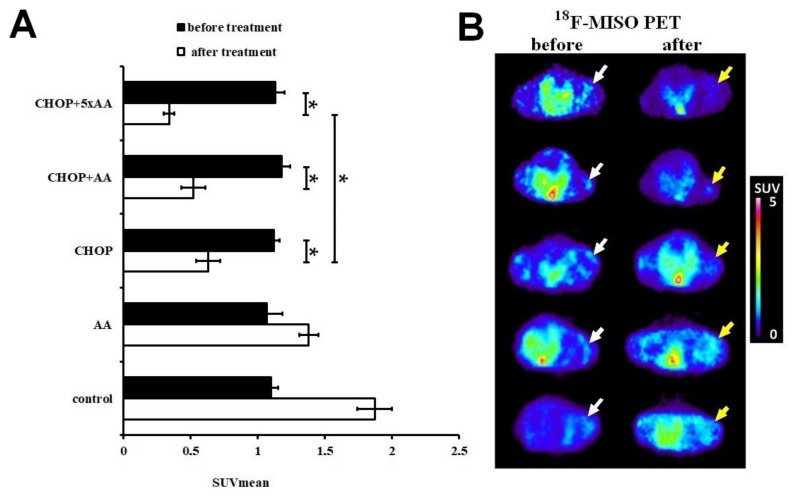
Comparative results of the in vivo PET analysis representing tissue hypoxia based on ^18^F-MISO SUVmean activity in A20/BalbC murine lymphomas before and after treatments. (**A**) Mean SUV values were similar in all untreated tumors (day 22, black columns). Five days after administration of the drug combinations (day 28, empty columns) a differential response was observed: general increase of SUVmean was measured in the untreated and AA groups, while a significant reduction in SUVmean was the result of CHOP and its combinations. AA adjuvant to CHOP further decreased ^18^F-MISO SUVmean values suggesting ongoing metabolic regression. (**B**) Representative decay-corrected miniPET images reflect the fading of tumor PET activity. Isotonic (0.9% NaCl) saline solution was used as a control. Animal horizontal cross sections are presented, central PET activity refers to ^18^F-MISO accumulation in the bladder. White arrows: A20 tumors before therapy, yellow arrows: the same A20 tumors after treatment. SUV: standardized uptake value. SUV data is presented as mean ± SD of at least 4 tumors/group. Significance level: *p* ≤ 0.05 (*).

To assess the effect of the antitumor combinations on hypoxia in the living tumor tissue, ^18^F-MISO PET imaging was performed on A20 tumor-bearing animals in vivo before and after treatment. According to the decay-corrected PET images obtained for all individual tumors, hypoxia was uniform and quite prominent prior to treatment (Figure 1A). This was confirmed by quantitative SUV data calculated for each treatment group (SUVmean values for control: 1.1 ± 0.05; AA: 1.07 ± 0.11; CHOP: 1.12 ± 0.04; CHOP+AA: 1.18 ± 0.06; CHOP+5xAA: 1.13 ± 0.07). However, the size of the labeled hypoxic areas within the tumor changed as a result of the treatments. The accumulation of ^18^F-MISO was observed after six days following treatment in the control tumors (SUVmean: 1.87 ± 0.13) and to a lesser degree in AA-treated tumors (SUVmean: 1.38 ± 0.07). In contrast, CHOP and CHOP+AA treatment combinations significantly reduced ^18^F-MISO uptake, indicating a massive decrease in the size of hypoxic areas in A20 tumors as a result of treatment response (post-treatment SUVmean values for CHOP alone: 0.63 ± 0.09; CHOP+AA: 0.52 ± 0.09; CHOP+5xAA: 0.34 ± 0.04). Moreover, while the effect of the single use of AA in combination with CHOP remained moderate, the CHOP+5xAA extended combination further reduced hypoxia related PET activity compared to CHOP alone in a significant manner (*p* < 0.05) (Figure 1A,B).

### 2.2. Tumor Volume

**Figure 2:**
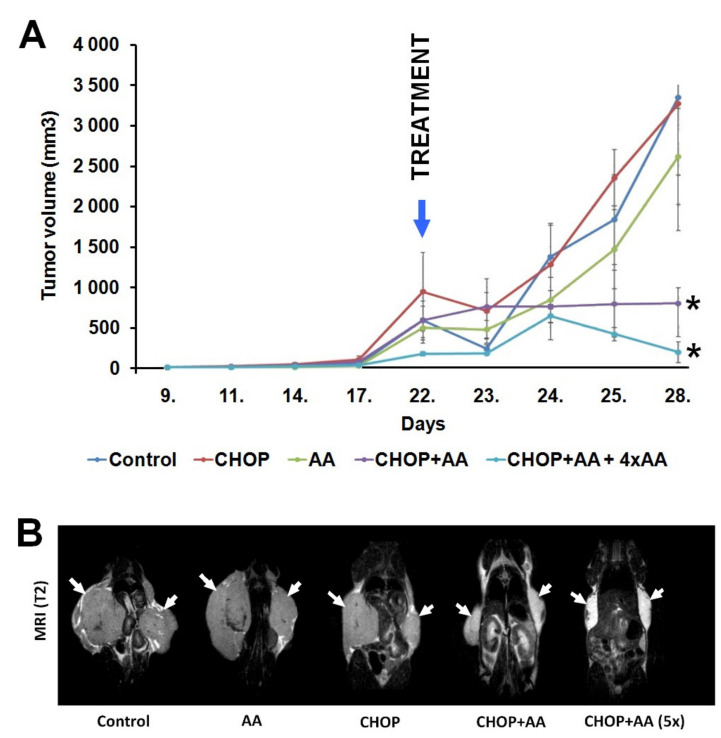
(**A**) Dynamics of the A20 tumor volume (mm^3^) in the different treatment groups. Control, CHOP and AA groups show a massive extension after day 23. In contrast, suppression of tumor growth was observed in the groups CHOP+AA and CHOP+AAx5. Significance level: *p* ≤ 0.05 (*) between the AA containing treatments and untreated or not AA containing treatment tumors at day 28. n = 3 mice/group; n = 2 A20 tumors/mouse. (**B**) Representative coronal T2-weighted MR images of A20 tumor-bearing mice at day 28. White arrows: A20 tumors.

In vivo tumor size was rigorously measured during the experiments and tumor volume was calculated for each site. Subcutaneous primary tumors were first detected and measured on day 9 following inoculation and reached a considerable size (200–850 mm^3^) by day 22. Treatment combinations resulted in highly different follow-up growth curves (Figure 2A). Tumors from untreated controls, and following AA alone and CHOP alone treatments, continuously expanded until the termination of the experiment on day 28 and reached tumor volumes of 3347.4 ± 962.5, 2615.2 ± 595.0, and 3269.0 ± 1568.8 mm^3^, respectively. In contrast, CHOP+AA combinations resulted significantly reduced tumor masses, with mean volumes of 797.1 ± 414.0 mm^3^ in the single CHOP+AA group and 191.7 ± 126.6 mm^3^ in the CHOP+AAx5 treatment group (both statistically significant at *p* ≤ 0.05). T2-weighted MR scans were performed to determine the inward spread of bilateral tumors on day 28. MRI images show that, at the end of the treatment, the A20 tumors of mice treated with AA-containing antitumor therapy (CHOP+AA and CHOP+AA (5x)) became symmetrically smaller than that of the control, AA, or CHOP treated tumors (Figure 2B).

### 2.3. General Histomorphology

**Figure 3:**
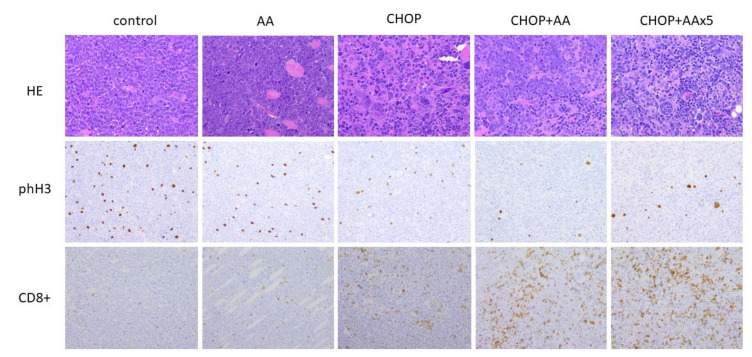
Intratumoral effects of CHOP and adjuvant AA determined by histology. Top row: General morphology of A20 lymphoma cells with giant cell transformation as a result of CHOP treatment (×40 magnification). Middle row: phospho-histone H3 immunolabeling demonstrating significant drop in cell proliferation activity following CHOP, CHOP+AA and CHOP+AAx5 treatments (×20 magnification). Bottom row: increase of CD8+ immunolabeling following CHOP+AA and CHOP+AAx5 compared to the control, AA and CHOP groups (×20 magnification).

A20 syngeneic lymphomas presented as bilateral subcutaneous infiltrative solid tissue masses at the site of the injection without obvious systemic dissemination. The tumor tissue histologically consisted of a densely-packed, large, monotonous cell mass with immature “blast” morphology, irregularly separated by foci of necrosis and fibrotic bundles. Compared to the untreated control samples AA treated tumors did not show considerable microscopic changes. However, all tumors exposed to the CHOP regimen, or any of its combinations, presented with extended foci of necrosis. Moreover, a significant proportion (up to 10%) of the CHOP treated cells featured with giant cell transformation typically characterized by 5–10-fold increase in size and multiple/multilobated cell nuclei, due to the expected cytokinesis inhibitory effect of vincristine (Figure 3, top row).

### 2.4. CAIX and CAXII Expression

**Figure 4:**
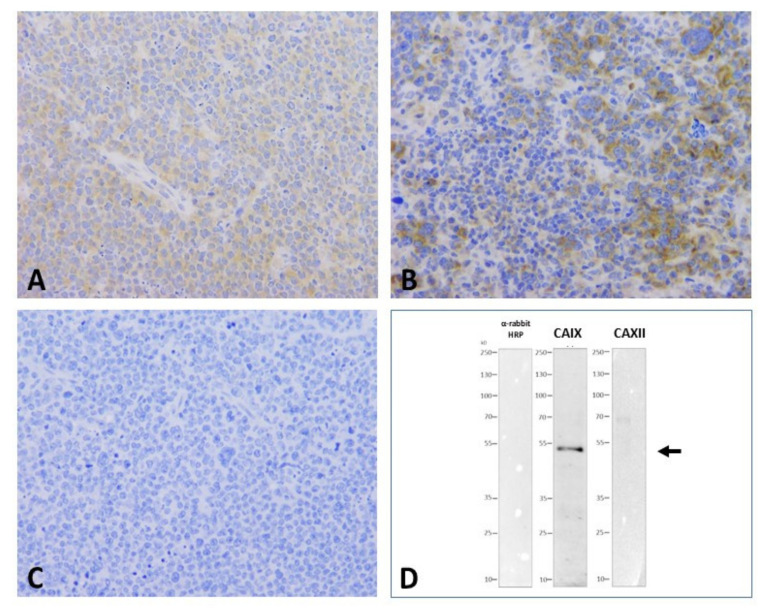
CAIX and CAXII presented by immunohistochemistry and Western blotting of A20 murine lymphoma grafts. Uniform weak cytoplasmic/membrane CAIX positivity of lymphoma cells (**A**); heterogeous IHC reaction with slight increase in the giant cell component following CHOP treatment, note negativity for the immune infiltrate (**B**); negative IHC staining for CAXII (**C**) (40× magnification). Western blot of A20 lymphoma cell lysate presenting a single specific band at 53–55 kDa for CAIX (center lane) and no signal for the CAXII protein (**D**, right lane, arrow). No unspecific signal by the detection system (based on anti-igG-HRP) applied in the absence of the primary antibodies was seen (negative control, left lane).

Expression of CAIX and CAXII as main cancer related carboanhydrases was systemically determined by IHC and WB analysis using established anti-CAIX and anti-CAXII antibodies. Control A20 lymphomas presented with a uniform CAIX cytoplasmic plus membrane positivity while virtually no CAXII expression was detected (Figure 4A,C). In general, CHOP and AA treatments did not influence the general pattern of CA expression. Perivascular areas appeared to retain positive staining even following massive cell death. Interestingly, giant cell foci presented with more intense membranous CAIX expression (Figure 4B). According to immunohistochemistry results, CAXII was not detectable in untreated A20 lymphomas and levels were not induced by any of the treatments (Figure 4C). WB data on A20 tumor cell lysates using the same antibodies indicated the specific identification of the CAIX protein by the selected antibody clone and further, the absence of CAXII expression in the same A20 cells (Figure 4D).

### 2.5. Proliferative/Mitotic Activity

**Figure 5:**
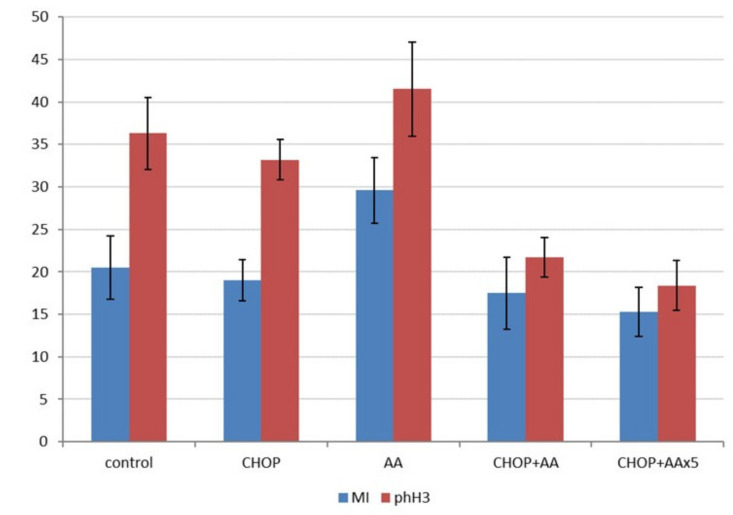
Mitotic index and phospho-histone 3 labeling index in A20 lymphoma treatment groups (y-axis represents mean counts/high-power field). Except of the series of the AA treatment only mild changes were stated when mitosis counting was applied (left columns). phH3 IHC, specifically detecting Ser10 phosphorylated histone3 required for mitotic entry in dividing cells, presented higher mitotic numbers and a statistically significant decrease in cell division activity in the CHOP+AA and CHOP+AAx5 treated tumors (right columns, *p* < 0.05).

Further to the in vivo tumor growth characteristics, the effects of the treatments were compared by determining the cell proliferation activity. Representative histological sections prepared from the final tumor samples were used to count mitotic figures following HE staining, and more specifically, phospho-histone 3 (Ser10) labelled mitotic cells, highlighted by IHC (Figure 3, middle row). Mitotic counts (mitotic index) were in the range of 15.3–29.6/hpf, with the highest values in the AA treated group (significantly different from all other treatment groups, *p* < 0.05). The mitotic index was not statistically different when the untreated control, CHOP, as well as CHOP+AA combinations were compared (Figure 5, left columns). In contrast, phospho-H3 IHC indicated a significant decrease in positive events the samples treated with adjuvant CHOP+AA or CHOP+AAx5 (mean range 21.7 ± 5.1 and 18.4 ± 2.9). These proved to be statistically lower compared to the control, CHOP, and AA groups (mean 36.3 ± 4.22; 33.2 ± 2.35 and 41.5 ± 5.5, respectively) (*p* < 0.05) (Figure 5, right columns). Extended AAx5 treatment did not result a significant change in the phH3 labeling compared to the single AA dose adjuvant to CHOP (*p* = 0.19).

### 2.6. T-cell Immune Infiltrate

**Figure 6:**
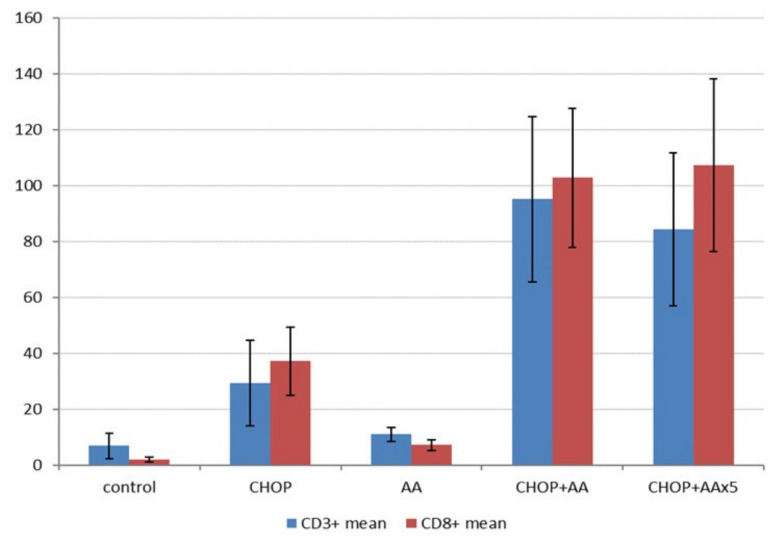
Results on T-cell infiltrates determined by immunohistochemistry in A20 lymphomas following different treatment combinations (Y-axis represents mean counts/high-power field). CD3+ (left columns) and CD8+ counts (right columns) changed in parallel. Treatment by CHOP alone resulted in a significant increase in the immune infiltrate (*p* < 0.05), which was strongly intensified by the adjuvant AA treatment (*p* < 0.05 for both values). Extended AA (AAx5) did not further improve the extent of lymphocytic infiltration (*p* = 0.188 and 0.32).

The A20/BalbC syngenic experimental setup provided a unique opportunity to determine basic tumor- host immune interactions. Immunohistochemistry of CD3+ and CD8+ T-cell infiltrate reflected remarkable differences in association with the combined treatments (Figure 3, bottom row). As expected, the amounts of CD3 and CD8 positive T-cells changed principally in parallel (Figure 6). Minimal infiltrates were demonstrated in the control lymphoma samples (CD3+ mean 6.9 ± 4.5/hpf, CD8+ mean 1.9 ± 0.7/hpf), which showed a considerable increase following single AA treatment (CD3+ mean 10.9 ± 2.9/hpf, CD8+ mean 7.1 ± 2.0/hpf, both *p* < 0.05). The regular CHOP treatment significantly (4–10x) increased the amount of T-cell infiltrate compared to the untreated control tumors (CD3+ mean 29.3 ± 15.2/hpf, CD8+ mean 37.2 ± 12.2/hpf, both *p* < 0.05). Moreover, combinations of CHOP and AA raised the amount of the immune infiltrate with an order of magnitude. CHOP+AA treated samples presented with mean CD3+ counts of 95.2 ± 29.5 (12x the value of the untreated control, 3x of the standard CHOP treatment) and CD8+ counts of 102.8 ± 24.8 (53x the value of the untreated control, 3x of the standard CHOP treatment, each *p* < 0.005). CHOP+AAx5 treatment resulted in mean CD3+ counts of 84.3 ± 27.3 and CD8+ counts of 107.4 ± 30.8/hpf, not significantly different from the single dose AA in combination (*p* = 0.188 and 0.32, respectively). Most importantly, immune infiltrates due to any of the CHOP+AA or CHOP+AAx5 adjuvant therapies proved to be significantly higher compared to that of the control, CHOP alone, as well as the AA alone treated groups (each *p* < 0.005) (Figure 6).

## 3. Discussion

The potential of CAs, and more specifically cancer related CAIX and CAXII as biological targets for anti-tumor therapy, has been intensively studied in the past two decades [7,17,19,21]. As the central paradigm, the inhibition of their pH neutralization effect effectively contributes to tumor cell damage by maintaining intracellular acidosis. Combinations of CA-inhibitors with other anticancer agents (histone deacetylase inhibitors, alkylating agents, antimetabolite nucleosides, angiogenesis inhibitors, and immune checkpoint inhibitors) have been evaluated and found to act synergistically [31]. Consistent with this theory, several treatment combinations, based on the use of small molecule CA inhibitors (including AA) were experimentally tested [18,22,23,24].

The expression of CAs due to hypoxic stress was reported in a series of aggressive lymphoma cell lines [27]. The increase of CAIX but not CAXII expression was associated with tissue necrosis and inferior clinical outcome in human lymphatic neoplasia, especially in progressive forms of classical Hodgkin-lymphoma [28,29]. However, attempts made for the therapeutic utilization of CAIX in lymphoma have not been reported so far. In our current experiments, directed to A20/BalbC lymphoma, we aimed to study the principle utility of CA inhibition in combination with the conventional anti-lymphoma regimen CHOP and decided to use AA for simplicity reasons.

The unique A20 syngeneic model, in contrast to many others, represent a close to natural scenario as neoplastic cells are seeded in the same BalbC mouse strain they originate from. This, further to the optimal growth conditions in particular enables the generation of anti-tumor immune interactions as the immunity of the host animal is intact. During our experiments, A20 B-lineage lymphoma cells grew intensively and formed considerable subcutaneous tumors after approx. 10–15 days following inoculation as expected. A20 lymphoma presented all characteristic features of high-grade B-cell lymphomas in terms of macro- and microscopic morphology and growth characteristics. As described, the obvious sensitivity of the in vivo tumors to the conventional CHOP regimen provided an optimal model for the study of treatment combinations [30]. In our practice, giant cell transformation, aberrant mitotic figures, apoptosis, and extended foci of necrosis clearly indicated the proper delivery and a direct cytotoxic effect in all CHOP treated samples. It was of particular importance that the profound analysis of the A20 lymphoma tissue provided evidence of significant carbonic anhydrase IX protein expression in these tumors, demonstrated by both IHC and WB. This was not the case for CAXII, the expression of which could not be proven, in agreement with previous findings [27,32]. Although the contribution of further members of the carbonic anhydrase family is not subsidiary, isoenzyme profiling was not in the scope of our current studies.

The in vivo effect of adjuvant AA together with the CHOP treatment in A20 lymphomas was obvious. The animals tolerated AA alone or in combination extremely well. Compared to the untreated tumors in control animals, administration of AA alone did not result in general drop of the tumor mass, despite of slight decrease of 18F-MISO PET activity (hypoxia) and increase in cell proliferation. These effects could be explained with the perfusion enhancing effect of AA through vasodilatation, as reported [33]. To our satisfaction, however, the tumor-reductive effect of the conventional CHOP regimen could be significantly intensified with a simultaneous single dose administration of AA. The improvement could be followed at many levels. We observed a shrinkage and delayed recovery of the lymphoma tumor mass due to both treatment combinations CHOP+AA or CHOP+AAx5 after four days. In line with this, in vivo ^18^F-MISO PET imaging presented a prominent decrease in tumor size and tissue hypoxia related activity, which could be intensified by the five-day extended AA dosage following a single CHOP bolus. In vivo functional PET findings were further supported by the dynamics of the tumor volume and cell proliferation data and were in good agreement with earlier findings [34,35].

The in vivo changes could be completed with several important observations following tissue-based studies. Histomorphological measurement of the cellular kinetics (especially by the use of anti-phospho-H3 immunohistochemistry) reflected a significant suppression of cell proliferation due to the combined AA treatments compared to the conventional CHOP protocol. Moreover, AA combination treatments massively increased the amount of intratumoral CD3+ and CD8+ T-cell infiltrates, exceeding the values of the conventional CHOP by approximately three times. Interestingly, extended administration of AA over four additional days following the therapy combination (CHOP+AAx5) did not have a strong effect on the histologically measured lymphoma parameters and the response remained significant, but comparable with the single dose CHOP+AA treatment.

Most of the effects observed in A20 lymphomas can be associated with the CA-inhibitory activity of AA. We assume that CAIX takes a leading role in this effect while, according to our data, CAXII does not seem to have a specific role in A20 cells. CAIX is particularly effective to restore the reduced intracellular pH upon metabolic stress and thus, contributes to tumor survival. In addition, the acidification of the tumor microenvironment gains special importance for both drug availability and tumor-host immune interactions. Tumor derived acidosis, generally induced by diverse pH-regulatory mechanisms, is now considered as a strong immune modulatory feature which interacts with therapy response [36,37,38]. Moreover, therapeutic neutralization of microenvironmental pH may directly improve the effect of immunotherapies, and thus was defined as a future direction of drug development [39,40].

Our histology-based results indicate to an important effect of CA inhibitor AA on the measure of intratumoral immune cell infiltrate in A20 lymphoma. According to our data the combination of AA with the conventional CHOP treatment not just increased the lethality of the cytotoxic regimen but also significantly intensified the physiological anti-tumor T-cell reaction, as represented by the amount of the host animal derived CD3+/CD8+ tumor infiltrating lymphocytes (TILs). The combination of AA with selected cytotoxic drugs was already reported to be effective in diverse settings [22,23,24]. However, there are currently no data available on the immune stimulatory effect of CA-inhibition to be exploited in antibody or immune-checkpoint mediated regimens. Intratumoral cytotoxic T-cell interactions are supposed to be a major requirement for proper response to immunotherapies and the number of TILs became a prognostic indicator in clinical setting. Our present data suggest that the intensity of the CD3+/CD8+ infiltrate can be stimulated by the simultaneous application of the prototypic CA-inhibitor acetazolamide. Although the function of CAIX, and thus the favorable effect of its inhibition, seems to be plausible, further studies including knock-out and silencing experiments are required to learn about the role of CA and CA-inhibition on the tissue microenvironment in lymphoma.

Importantly, the potentially beneficial action of AA in the fight against lymphomas can be highly variable. Beside the CA-inhibitory effect the redistribution of vascular perfusion (favoring local vasodilatation) may also contribute to enhanced delivery of cytotoxic substances and immune effectors [33]. Therefore, the striking response we observed following the combined CHOP+AA treatment may be the beneficial sum of several intra- and extracellular mechanisms. These include, but are not limited to i) the direct increase of cancer cell lethality, ii) the suppression of cancer cell invasiveness, iii) the delayed matrix degradation by cancer specific acidic proteinases, iv) the indirect support of therapeutic drug and antibody delivery, and v) the enhanced tumor cell accessibility for anti-tumor effector immune cells.

In summary, we provide early experimental results supporting the advantage of CA-inhibitors as adjuvants administered together with anti-tumor combinations in lymphoma. CHOP—now supplemented with anti-CD20 antibody rituximab—is a standard and effective treatment regimen for aggressive forms of the disease. In the A20/BalbC syngeneic lymphoma model, a single dose of CHOP measurably suppresses tumor progression which is improved by the additional dosage of CA-inhibitor AA. This is the first report presenting the potential efficacy of a CA-inhibitor in combination with a conventional chemotherapy regimen in lymphoma. Moreover, our data indicate a strong interplay between CA-inhibition and anti-tumor immune infiltrates. Although the function of CAs, and thus the favourable effect of CA inhibitors, seems to be plausible, further studies including detailed genomic, knock-out, and gene silencing experiments are required to learn about the specific role in the composition of the tissue microenvironment of lymphomas and other malignancies.

## 4. Materials and Methods

### 4.1. In Vitro Culturing of A20 Murine Lymphoma Cells

A20 murine lymphoma cell line was purchased from the American Type Culture Collection (ATCC, TIB-208TM). A20 cells were cultured at 5% CO_2_, 37 °C in RPMI-1640 Medium with L-glutamine and high-glucose (GIBCO Life Technologies) supplemented with 10% Fetal Bovine Serum (FBS, GIBCO Life technologies) and 1% Antibiotic and Antimycotic (Sigma-Aldrich). For in vitro studies and tumor induction, the cells were used at 85% confluence and the viability of the cells was always higher than 90%, as assessed by the trypan blue exclusion test.

### 4.2. A20/BalbC Syngenic Murine Lymphoma Model

BALB/c mice (12-week-old male mice, purchased from Innovo Ltd., Hungary) were used for the in vivo experiments. Mice were housed under conventional conditions in IVC cage system (Techniplast, Italy) at a temperature of 26 ± 3 °C, with 52 ± 10% humidity and artificial lighting with a circadian cycle of 12 h. Semi-synthetic diet (Akronom Ltd., Budapest, Hungary) and drinking water were available ad libitum to all the animals. Laboratory animals were kept and treated in compliance with all applicable sections of the Hungarian Laws, the regulations of the European Union and the Code of Practice for the Housing and Care of Animals Used in Scientific Procedures Act 1986.

Lymphomas were inoculated by subcutaneous injection of viable A20 cells (5 × 10^6^ cells in 100 µL 0.9% NaCl) to both shoulder area of the BALB/c mice according to Bascuas et al. [30]. Animals were controlled regularly, and tumor growth dynamics was documented. Day 0 for CHOP/AA treatment was defined by reaching a mean tumor volume of approximately 500 mm^3^.

### 4.3. Animal Treatment and Drugs

Treatment of A20 tumor-bearing mice was started 23 ± 1 days after tumor cell inoculation at a tumor size of 8–10 mm (n = 20). Tumor-bearing animals were randomized into 5 groups (4 mice/group) as follows: group 1: control group was treated only with 0.9% NaCl (100 µL) intravenously; group 2 was treated once intravenously with acetazolamide (AA, 160 mg/kg); group 3 was treated once intravenously with the CHOP cocktail consisted of cyclophosphamide (100 mg/kg), doxorubicin (6 mg/kg), vincristine (0.1 mg/kg) and dexamethasone (0.2 mg/mL); group 4 was treated once intravenously with the combination of CHOP+AA; group 5 was treated once intravenously with the CHOP+AA combination and 4 more times with AA only on four consecutive days.

Changes in tumor size and general condition of the mice were checked daily. Animals were sacrificed on day 28 following treatment. All tumors/sites of inoculation were dissected, and tissue samples were taken for histology and protein studies (−80 °C frozen samples).

### 4.4. In Vivo MRI and PET Imaging of Tumor Hypoxia

Tumor hypoxia imaging was performed using a small animal PET device before (23 ± 1 days after tumor cell inoculation) and after (27 ± 1 days after tumor cell inoculation) anticancer therapy. A20 lymphoma-bearing animals were injected with 9.3 ± 0.4 MBq of hypoxia specific ^18^F-MISO ([^18^F] Fluoromisonidazole) via the lateral tail vein before and after anticancer treatments. Then, 90 min after radiotracer injection, mice were anaesthetized by 2% isoflurane (Forane) with a dedicated small animal anesthesia device and static PET scans (20 min acquisition time) were acquired using the preclinical MiniPET-II scanner. T2-weighted MR scans (3D multiecho, repetition time (TR) = 3000 ms, acquisition matrix = 256 × 201 × 20 and image spatial resolution = 0.532 × 0.532 × 2 mm^3^) were performed using the Philips Achivea 3T scanner to determine the inward spread of A20 tumors on day 28 following treatment.

### 4.5. Quantitative PET Data Analysis

Quantitative radiotracer uptake was expressed in terms of standardized uptake value (SUV), SUV = (VOI activity (Bq/mL))/(injected activity (Bq)/animal weight (g)), assuming a density of 1 g/mL. Volumes of interest (VOI) were manually drawn around the edge of the tumor activity by BrainCad image analysis software.

### 4.6. Tissue-based Analysis

Multiple tumor tissue samples of approx. 6 × 6 mm size were taken and put immediately in 10% buffered formalin for histological studies. Following tissue processing formaldehyde fixed and paraffin embedded (FFPE) samples were sectioned and stained for conventional HE and immunohistochemistry (IHC) according to routine laboratory protocols. CAIX antigen was demonstrated by the rabbit polyclonal antibody clone NB100-417 (Novus Biologicals, Littleton, CO) at a final dilution of 1:2000. For CAXII IHC the rabbit polyclonal antibody clone PA5-52608 (Invitrogen-Thermo Fisher, Life Sciences, Budapest) was used at final dilution of 1:1000. To highlight mitotic activity specifically an antibody specific for phospho-histone 3 was used (pH3 phospho S10, clone ab5176, AbCam, Cambridge, UK) following antigen retrieval at pH6.0 at a final dilution of 1:4000). Tumor infiltrating T-lymphocytes were visualized by anti CD3 (clone SP7, Thermo-Fisher, Life Sciences, Budapest, Hungary, dilution 1:200) and anti CD8 (clone ab209775, Abcam, Biokastel, Budapest, Hungary, dilution 1:2000) antibodies. IHC stainings were done using the Envision+ HRP-labeled polymer detection system (no. K4002, Dako-Agilent, Budapest, Hungary).

The classical mitosis index was determined using representative H&E sections as the sum of well discernible mitotic figures counted within the lymphoma in 10 randomly selected high-power fields (40× magnification). phH3 positive mitotic cells were identified on the basis of a strong and dense immunopositive staining of chromatin clumps. The total of phH3 positive M-phase cells was counted within the tumor in 10 randomly selected HPFs (40× magnification).

CD3+ and CD8+ T-cell counting was done using the Leica microscopic imaging device (Leica LASV4.13 software). Microscopic slides were uniformly projected on the screen using the 40× objective. Individual images of tissue areas fully covering the field of view were counted for immunopositive events, results were given as mean positive events/field of view. At least 10 representative fields were counted.

### 4.7. Western Blotting and Validation of CAIX and CAXII Specific Staining

A20 lymphoma tissue was minced with a bead beater (TissueLyser II, Qiagen). Cells were lysed in RIPA buffer (50 mM Tris, 150 mM NaCl, 0.1% SDS, 1% TritonX 100, 0.5% sodium deoxycolate, 1 mM EDTA, 1 mM Na_3_VO_4_, 1 mM NaF, 1 mM PMSF, protease inhibitor cocktail). Protein isolation, SDS-PAGE, and Western blotting were performed according to Nagy et al. [41]. Protein samples (10–40 µg) were separated on 10% SDS polyacrylamide gels and electrotransferred onto nitrocellulose membranes. After blocking for 1 h with TBST containing 5% BSA, the membranes were incubated with the primary antibodies against CAIX and CAXII overnight at 4 °C (dilution: 1:1000) or with TBST. After washing with 1 x TBST solution, the membranes were probed with IgG HRP conjugated secondary antibody (Cell Signaling Technology, Inc. Beverly, MA, 1:2000). Bands were visualized by enhanced chemiluminescence reaction (SuperSignal West Pico Solutions, Thermo Fisher Scientific Inc., Rockford, USA).

### 4.8. Statistical Analysis

Significance was calculated by two-way ANOVA, Student’s *t*-test (two-tailed), and Mann–Whitney U-test and the significance level was set at *p* ≤ 0.05 unless otherwise indicated. Data are presented as mean ± SD.
